# Preparation and Characterization of Bioplastic-Based Green Renewable Composites from Tapioca with Acetyl Tributyl Citrate as a Plasticizer

**DOI:** 10.3390/ma7085617

**Published:** 2014-08-04

**Authors:** Chi-Hui Tsou, Maw-Cherng Suen, Wei-Hua Yao, Jen-Taut Yeh, Chin-San Wu, Chih-Yuan Tsou, Shih-Hsuan Chiu, Jui-Chin Chen, Ruo Yao Wang, Shang-Ming Lin, Wei-Song Hung, Manuel De Guzman, Chien-Chieh Hu, Kueir-Rarn Lee

**Affiliations:** 1 Department of Materials Science and Engineering, National Taiwan University of Science and Technology, No. 43, Sec. 4, Keelung Rd., Da’an Dist., Taipei 10607, Taiwan; E-Mails: keymykimo@gmail.com (C.-Y.T.); schiu@mail.ntust.edu.tw (S.-H.C.); 2 Research and Development Center for Membrane Technology, Department of Chemical Engineering, Chung Yuan University, Chung-Li 32023, Taiwan; E-Mails: maweidegz@gmail.com (M.D.G.); cchu@cycu.edu.tw (C.-C.H.); krlee@cycu.edu.tw (K.-R.L.); 3 Department of Materials and Textiles, Oriental Institute of Technology, Pan-Chiao 22064, Taiwan; E-Mails: angelayao@mail.oit.edu.tw (W.-H.Y.); fc011@mail.oit.edu.tw (J.-C.C.); steven5202001@yahoo.com.tw (R.Y.W.); fc013@mail.oit.edu.tw (S.-M.L.); 4 Department of Creative Fashion Design, Taoyuan Innovation Institute of Technology, Jhongli 32091, Taiwan; E-Mail: sun@tiit.edu.tw; 5 Department of Chemical and Biochemical Engineering, Kao Yuan University, Kaohsiung County 82151, Taiwan; E-Mail: jyeh@mail.ntust.edu.tw; 6 Faculty of Materials Science and Engineering, Hubei University, Wuhan 430062, China; E-Mail: cws1222@cc.kyu.edu.tw

**Keywords:** biodegradable, poly(lactic acid) (PLA), tapioca, methylenediphenyl diisocyanate (MDI), acetyl tributyl citrate (ATBC)

## Abstract

Granular tapioca was thermally blended with poly(lactic acid) (PLA). All blends were prepared using a plasti-corder and characterized for tensile properties, thermal properties and morphology. Scanning electron micrographs showed that phase separation occurred, leading to poor tensile properties. Therefore, methylenediphenyl diisocyanate (MDI) was used as an interfacial compatibilizer to improve the mechanical properties of PLA/tapioca blends. The addition of MDI could improve the tensile strength of the blend with 60 wt% tapioca, from 19.8 to 42.6 MPa. In addition, because PLA lacked toughness, acetyl tributyl citrate (ATBC) was added as a plasticizer to improve the ductility of PLA. A significant decrease in the melting point and glass-transition temperature was observed on the basis of differential scanning calorimetry, which indicated that the PLA structure was not dense after ATBC was added. As such, the brittleness was improved, and the elongation at break was extended to several hundred percent. Therefore, mixing ATBC with PLA/tapioca/MDI blends did exhibit the effect of plasticization and biodegradation. The results also revealed that excessive plasticizer would cause the migration of ATBC and decrease the tensile properties.

## 1. Introduction

Poly(lactic acid) (PLA) resins are well-known biodegradable, linear aliphatic thermoplastics, which can be produced from renewable resources [1,2]. They are one of the most promising polymers of their family [3] and are highly accepted as biomedical materials, because of their biocompatibility and good mechanical properties [4,5]. However, their brittleness, slow crystallization and being easily hydrolyzed limit their usage in many applications. In fact, it is difficult to use them for film blowing or extrusion, unless their moisture content and processing conditions are carefully controlled. Moreover, their price competitiveness in the biodegradable plastic market is an essential attribute that cannot be ignored. The most effective approach to reduce the capital cost of PLA is to use fillers. Cost-effective reinforcements are organic renewable resources [6], flax [7,8,9], sisal [10], lyocell [11], short abaca [12], jute [13], bamboo [14], paper pulp [15,16], pineapple [17], Cordenka [18], microcrystalline cellulose [19], and kenaf [20]. Starch is attractive because of its low cost, renewability, biodegradability, low density and non-abrasiveness. A lot of studies on the blending of PLA/starch [21,22,23,24,25,26,27,28,29,30], such as wheat starch [21,24,30], corn starch [26,27,29,31] and cassava starch [23], have been researched. Tapioca was used as a filler in [32], because it is cheap, and fewer reports compared it with other starches. However, the poor interfacial adhesion between the filler and the polymer generally leads to composites with worse mechanical properties. Surface and bulk modifications of the filler and/or matrix are necessary to increase the interfacial compatibility between the hydrophilic filler and the hydrophobic PLA matrix. Some studies used methylenediphenyl diisocyanate (MDI) as a compatibilizer to improve the compatibility between PLA and starch [21,30] or between PLA and rice husk [33]. These biopolymers are successfully prepared with starch or rice husk blends using MDI as a coupling agent. Copolymerization or blending PLA with other polymers [34,35,36,37,38,39,40,41,42,43] or compounds (e.g., plasticizers) [44,45,46,47,48,49] was proven to be a feasible way to improve its processability in film products for extrusion and/or film blowing. In this study, tapioca was used as a filler to reduce the cost of PLA products; MDI was used as a coupling agent to enhance the interfacial compatibility between PLA and tapioca; and ATBC was used as a plasticizer to improve the processability, flexibility and ductility of glassy PLA/tapioca composites. The result of this study could provide a database useful for the design and manufacture of biodegradable materials.

## 2. Results and Discussion

### 2.1. Tensile Property

**Figure 1:**
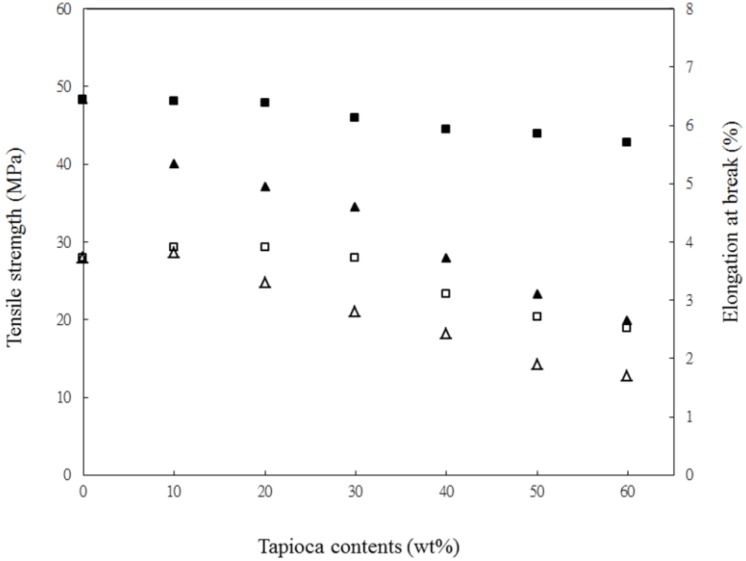
Tensile strength *vs.* tapioca content for (▲) PLA/tapioca and (■) PLA_/_tapiocaMDI_0.5_; elongation at break *vs.* tapioca content for (△) PLA/tapioca and (□) PLA_/_tapioca*_y_*MDI_0.5_.

The tensile strength (σ_f_) and elongation at break (ε_f_) of PLA*_x_*Tapioca*_y_* and PLA_90_Tapioca_10_MDI are plotted in Figure 1. The σ_f_ of PLA is 48.3 MPa. After blending PLA with tapioca, PLA*_x_*tapioca*_y_* specimens revealed a substantial reduction in σ_f_ and ε_f_. For example, the σ_f_ of PLA*_x_*Tapioca*_y_* specimens was reduced from 48.3 to 17.5 MPa as the tapioca content increased from 0 to 60 wt%. When MDI was added, the σ_f_ of the PLA*_x_*tapioca*_y_* specimens was improved; the σ_f_ of PLA_40_Tapioca_60_MDI was 42.6 MPa, whereas that of PLA_40_Tapioca_60_ was 25.1 MPa. This improvement in σ_f_ is due to the increase in the PLA*_x_*Tapioca*_y_* interfacial adhesion, as a result of the formation of urethane linkages between MDI and PLA, as well as those between MDI and tapioca, because MDI acts as a coupling agent [21]. The ε_f_ for all samples are between 1% and 4%; thus, their toughness is similar.

**Figure 2:**
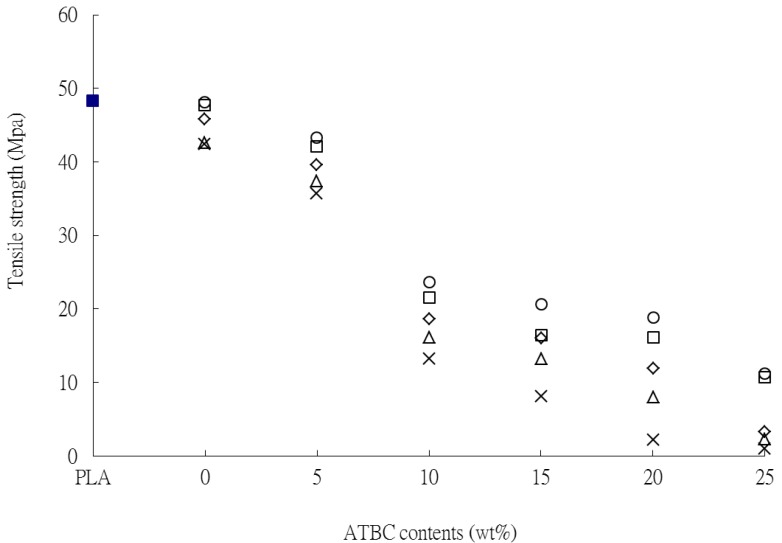
Tensile strength *vs.* ATBC content for (■) PLA; (○) PLA_90_tapioca_10_MDI_0.5_; (□) PLA_80_tapioca_20_MDI_0.5_; (◇) PLA_70_tapioca_30_MDI_0.5_; (△) PLA_60_tapioca_4__0_MDI_0.5_; and (×) PLA_50_tapioca_50_MDI_0.5_.

**Figure 3:**
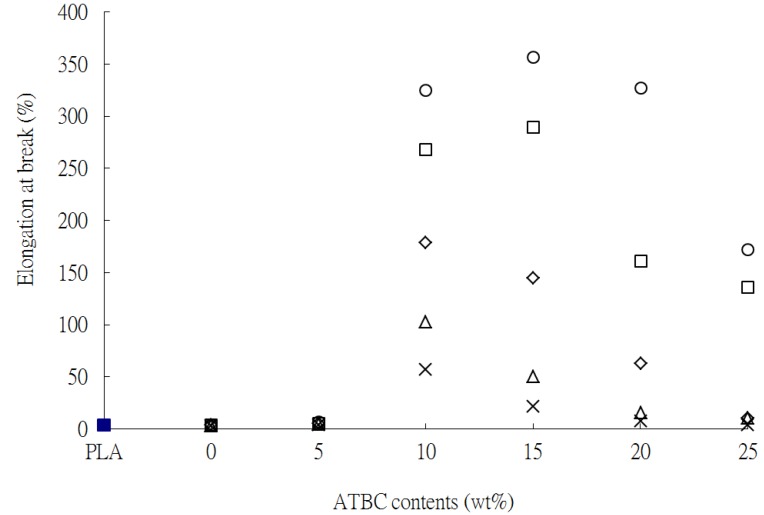
Elongation at break *vs.* ATBC content for (■) PLA; (○) PLA_90_tapioca_10_MDI_0.5_; (□) PLA_80_tapioca_20_MDI_0.5_; (◇) PLA_70_tapioca_30_MDI_0.5_; (△) PLA_60_tapioca_4__0_MDI_0.5_; and (×) PLA_50_tapioca_50_MDI_0.5_.

The σ_f_ and ε_f_ of PLA and PLA*_x_*tapioca*_y_*MDI specimens as a function of ATBC are shown in Figure 2 and Figure 3, respectively. With increasing plasticizer content, a common trend is shown by all series investigated: the σ_f_ decreases, whereas the ε_f_ increases. The σ_f_ of PLA*_x_*tapioca*_y_*MDI was reduced much more significantly after ATBC was added; the σ_f_ value of PLA_50_tapioca_50_MDI was reduced from 42.3 to 0.9 MPa, as the ATBC content was increased from 0 to 25 wt% (see Figure 2). This substantial reduction is due to the tapioca of PLA*_x_*tapioca*_y_*MDI that could not be plasticized by ATBC. In addition, the ε_f_ of PLA_90_tapioca_10_MDI, PLA_80_tapioca_20_MDI, PLA_70_tapioca_30_MDI, PLA_60_tapioca_40_MDI and PLA_50_tapioca_50_MDI approaches the maximum value at 357.3%, 289.7%, 178.8%, 102.5% and 56.3%, respectively, as the ATBC content reaches an optimum value of 10 or 15 wt%. The ε_f_ of the PLA*_x_*tapioca*_y_*MDI specimens was reduced as the ATBC content increased from 10 or 15 wt%. These results clearly suggest that the relatively poor ductility of PLA*_x_*tapioca*_y_*MDI was improved after blending proper amounts of ATBC with the PLA*_x_*Tapioca*_y_*MDI composites.

### 2.2. Fourier Transform Infra-Red Spectroscopy

**Figure 4:**
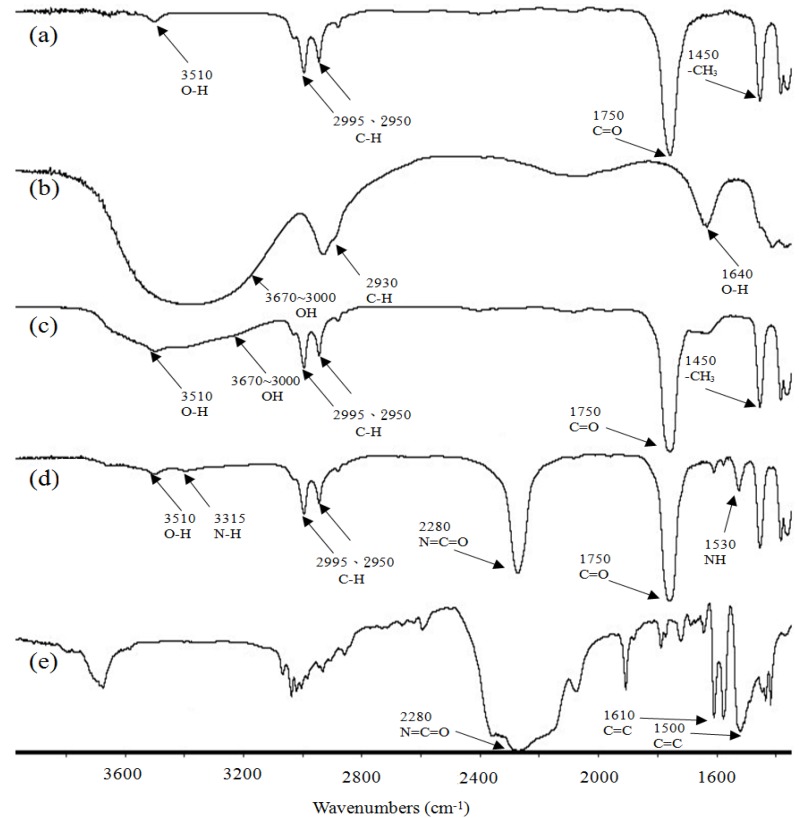
FTIR of: (**a**) PLA; (**b**) tapioca; (**c**) PLA/tapioca; (**d**) PLA/tapioca/MDI; and (**e**) MDI.

**Scheme 1:**
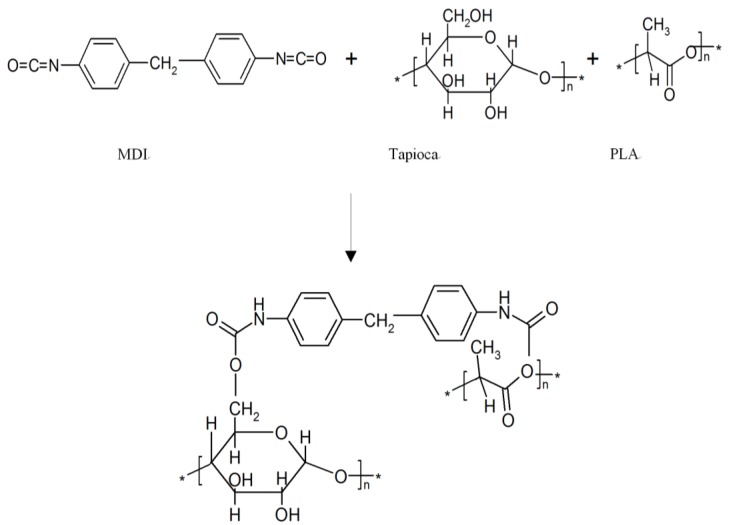
Possible reactions for PLA/tapioca/MDI composites.

Figure 4 illustrates typical FTIR spectra of PLA, tapioca, MDI, PLA*_x_*tapioca*_y_* and PLA*_x_*tapioca*_y_*MDI specimens. Four characteristic absorption bands centered at 1750, 2950, 2995 and 3510 cm^−1^, corresponding to the motions of C=O bending, C-H aliphatic stretching, C-H aliphatic stretching (doublet) and C-O-O-H stretching vibrations, respectively, were found in the spectrum of PLA (see Figure 4a). The FTIR spectra of PLA*_x_*tapioca*_y_* specimens, indicated in Figure 4c, are very similar to those of PLA; the four main absorption bands centered at 1750, 2950, 2995 and 3510 cm^−1^ were also found in the spectra of PLA*_x_*tapioca*_y_* specimens. The absorption bands around 3000 to 3670 cm^−1^ were the O-H stretching vibration of tapioca. The FTIR spectra of PLA*_x_*tapioca*_y_* specimens are very similar to those of PLA*_x_*tapioca*_y_*MDI (see Figure 4d). However, the aforementioned 3000 to 3670 cm^−1^ absorption bands originally shown in the FTIR spectra of PLA were gradually replaced by a newly developed absorption band centered at 3315 cm^−1^, which corresponds to the motion of the amine (N-H) stretching vibration. The disappearance of the 3000 to 3670 cm^−1^ bending absorption bands and the appearance of 3315 and 1550 cm^−1^ (N-H) stretching absorption bands are attributed to the reaction of the hydroxyl (O-H) groups of tapioca molecules with the urethane (N=C=O) groups of MDI and/or to the reaction of the carboxylic acid (C-O-O-H) groups of PLA molecules with the urethane groups of MDI during the melt-blending of PLA*_x_*tapioca*_y_* specimens. The possible mechanism for PLA/tapioca/MDI composites is shown in Scheme 1.

### 2.3. Morphology Analysis

**Figure 5:**
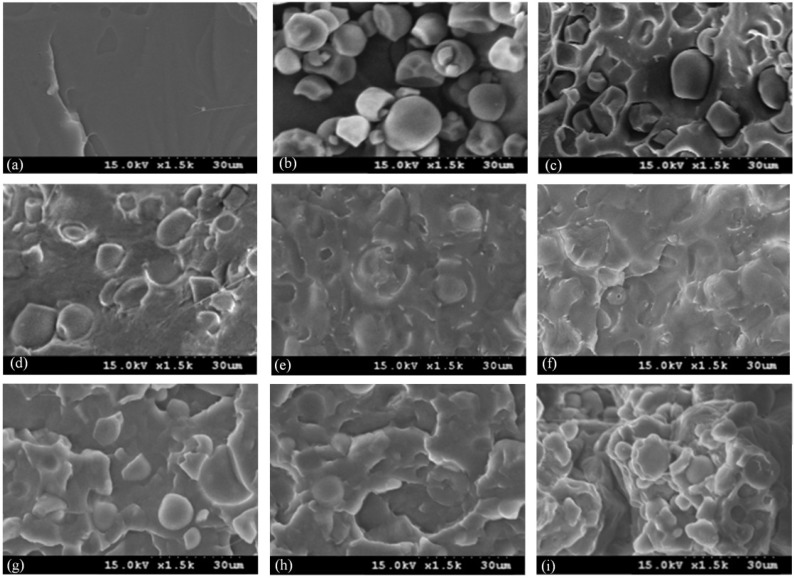
SEM for: (**a**) PLA; (**b**) tapioca; (**c**) PLA_7__0_tapioca_3__0_; (**d**) PLA_7__0_Tapioca_3__0_MDI_0.5_; (**e**) (PLA_7__0_tapioca_3__0_MDI_0.5_)_95_ATBC_5_; (**f**) (PLA_70_tapioca_30_MDI_0.5_)_90_ATBC_10_; (**g**) (PLA_7__0_tapioca_3__0_MDI_0.5_)_85_ATBC_15_; (**h**) (PLA_7__0_tapioca_3__0_MDI_0.5_)_80_ATBC_20_; and (**i**) (PLA_7__0_tapioca_3__0_MDI_0.5_)_75_ATBC_25_.

Typical SEM micrographs of (PLA_70_tapioca_30_MDI)*_a_*ATBC*_b_* specimens are shown in Figure 5. As shown in Figure 5a, a relatively brittle and smooth surface morphology was found for PLA. Tapioca is shown in Figure 5b. After blending PLA with tapioca, intervals between PLA and tapioca granules appeared (see Figure 5c) These results are similar to some reports on PLA/wheat starch [21] and PLA/corn starch blends [27,29]. This morphology is typical of incompatible blends, resulting in a poor tensile property, which is consistent with the result in Figure 1. After MDI was added, the compatibility of the PLA_70_tapioca_30_ specimen was improved (see Figure 5d). It shows excellent compatible morphologies, without the interval and voids associated with poor interfacial adhesion. The better compatibility of PLA_70_tapioca_30_MDI was due to the reaction of the hydroxyl groups of tapioca with the urethane groups of MDI and the reaction of the carboxylic acid groups of PLA with the urethane groups of MDI. The SEM micrographs of PLA_70_tapioca_30_MDI as a function of the increasing ATBC content (from 5 to 25 wt%) are shown in Figure 5e–h. The surface of PLA_70_tapioca_30_MDI was still smooth and without intervals when the ATBC content was 5 wt%. Furthermore, two phases can be seen after the ATBC content reaches 10 wt%, which is the threshold limit value of high ε_f_ for (PLA_70_tapioca_30_MDI)*_a_*ATBC*_b_* specimens (see Figure 3). With increasing ATBC content, more demarcated plastic-deformed PLA debris or fibrils were found on the surface of PLA_70_Tapioca_30_MDI (see Figure 5f–i). This was attributed to the increasing distance between PLA molecules, or the exudation of ATBC, and also to the deterioration of the interfacial adhesion between tapioca and PLA. Therefore, the σ_f_ of PLA*_x_*tapioca*_y_*MDI decreased significantly with increasing ATBC.

### 2.4. Differential Scanning Calorimetry

**Figure 6:**
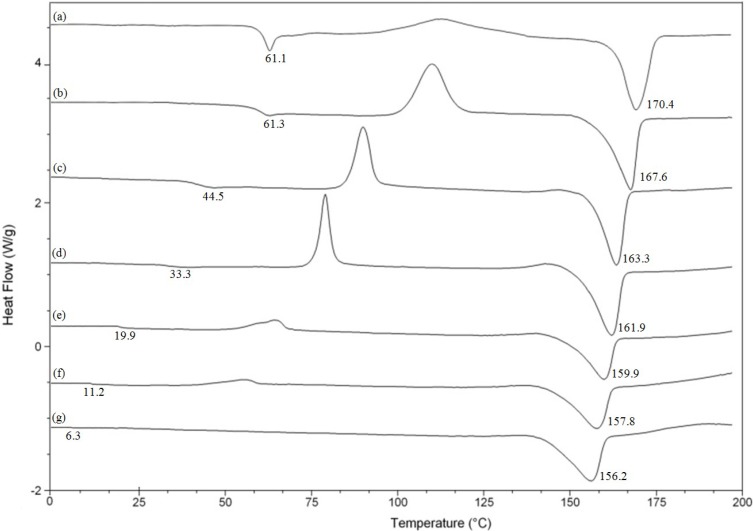
DSC for: (**a**) PLA; (**b**) PLA_7__0_tapioca_3__0_MDI; (**c**) (PLA_7__0_tapioca_3__0_MD)_95_ATBC_5_; (**d**) (PLA_70_tapioca_30_MDI)_90_ATBC_10_; (**e**) (PLA_7__0_tapioca_3__0_)_85_ATBC_15_; (**f**) (PLA_70_tapioca_3__0_MDI)*_a_*ATBC*_b_*;and (**g**) (PLA_70_tapioca_3__0_MDI)_75_ATBC_25_.

The thermal behavior of PLA and (PLA_70_tapioca_30_MDI)*_a_*ATBC*_b_* specimens was investigated. Figure 6 shows the DSC curves of PLA, the (PLA_70_tapioca_30_MDI)*_a_*ATBC*_b_* specimens and the plasticizer. The crystallization and melting enthalpies are identical, showing that PLA is totally amorphous after melt quenching. The DSC curves of PLA_70_tapioca_30_MDI show a single glass transition that decreases with increasing ATBC concentration (from 0 to 25 wt%). This result agrees with the data by Yeh *et al.* [50] and Baiardo *et al.* [51], who analyzed PLA/triacetin (TAc) and PLA/ATBC composites, respectively, over a more limited composition range (0%–30% TAc and ATBC). A decreasing *T*_g_ trend with increasing ATBC content is also shown in Figure 6, which is related to the cold crystallization and melting phenomena.

### 2.5. Water Absorption

**Figure 7:**
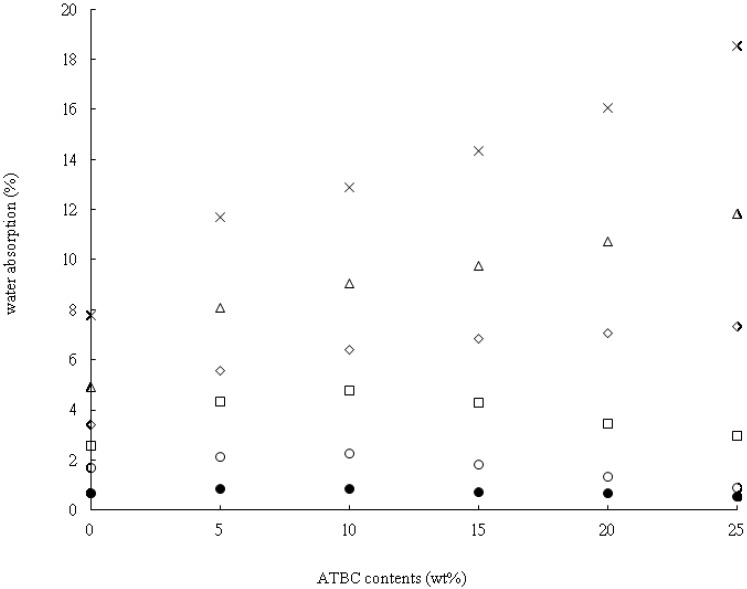
Water absorption *vs.* ATBC content for (●) PLA; (○) PLA_90_tapioca_10_MDI_0.5_; (□) PLA_80_tapioca_20_MDI_0.5_; (◇) PLA_70_tapioca_30_MDI_0.5_; (△) PLA_60_tapioca_4__0_MDI_0.5_; and (×) PLA_50_tapioca_50_MDI_0.5_.

Figure 7 presents data on the water absorption of the PLA*_x_*ATBC*_y_* specimens as a function of varying tapioca content. It shows a rising water absorption rate with increasing tapioca content at the same ATBC content. Water absorption rates were 0.58% to 18.57% when the tapioca content was from 0% to 50% at 25% ATBC content. This is due to the hydrophilic property of tapioca, causing the percentage of water absorption to increase. Furthermore, water absorption rates increased when ATBC was added; the water absorption rate of PLA_60_tapioca_40_ was 4.91% to 11.85% when the ATBC content was from 0% to 25%. ATBC is hydrophobic, but shows an interesting phenomenon of increasing moisture content. The trend of increasing water absorption is attributed to ATBC, which could enhance the free volume in PLA. This is evidenced by DSC analysis (see Figure 6): *T*_g_ decreased with increasing ATBC content. Therefore, the water molecule could be absorbed by PLA*_x_*tapioca*_y_* easily when the ATBC content increased the mobility of PLA molecules. The water adsorption for the lower tapioca content (10% to 30%) was first increasing and then decreasing. This might be due to slight exudation when the ATBC content is from 10% to 25%. It could be evidenced from the morphology analysis. The phase separation may be because of the exudation of ATBC; the phase separation worsens when the amount of ATBC was higher than 10% (See Figure 5f–i). However, when the tapioca content approached 40% and 50%, the ATBC might be absorbed by tapioca. Therefore, the exudation effect might not be apparent. On the basis of the results from the tensile property, morphology analysis and water absorption, the miscibility limits of ATBC content are suggested to be 10% for PLA*_x_*tapioca*_y_*MDI_0.5_ composites.

### 2.6. Enzymatic Hydrolysis

**Figure 8:**
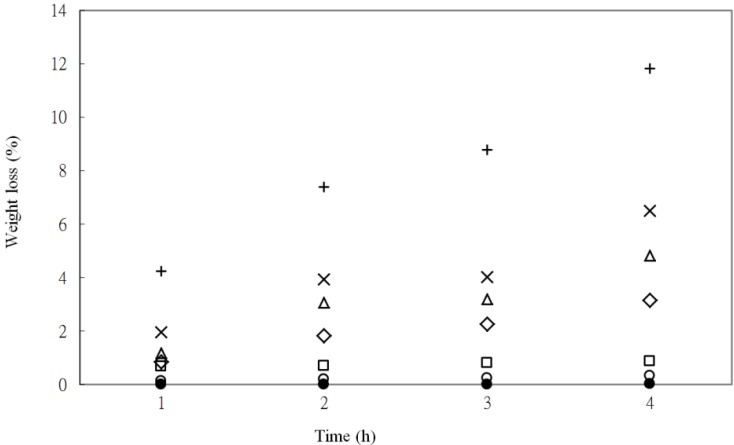
Weight loss for (●) PLA; (○) PLA_7__0_tapioca_3__0_MDI_0.5_; (□) (PLA_7__0_tapioca_3__0_MDI_0.5_)_95_ATBC_5_; (◇) (PLA_70_tapioca_30_MDI_0.5_)_90_ATBC_10_;(△) (PLA_70_tapioca_30_MDI_0.5_)_85_ATBC_15_; (×) (PLA_70_tapioca_30_MDI_0.5_)_80_ATBC_20_; and (+) (PLA_70_tapioca_30_MDI_0.5_)_75_ATBC_25_.

The weight loss of PLA_70_tapioca_30_MDI and (PLA_70_tapioca_30_MDI)*_a_*ATBC*_b_* at varying enzymatic hydrolysis time is indicated in Figure 8. It shows a common result: the increasing percentage of weight loss for all series, as the hydrolysis time increases. The weight loss of PLA_70_tapioca_30_MDI increased significantly from 0.33% to 11.82%, as the ATBC content increased from 0 to 25 wt% after 120 h of hydrolysis time. Enzymes could attack the molecules of PLA easily after ATBC was blended with PLA_70_tapioca_30_MDI. Furthermore, the interfacial adhesion between PLA and tapioca might deteriorate when ATBC was added. The weight loss increased significantly when the ATBC content was higher than 5%. The migration of ATBC occurred from 10% content. This conjecture of exudation was evidenced by the morphological analysis and water absorption.

## 3. Experimental Section

### 3.1. Materials and Preparation

PLA resin, with a trade name of Nature Green 4032D, was obtained from Cargill-Dow. Tapioca was purchased from United Global Agencies (Bangkok, Thailand). Before melt-blending, PLA and tapioca were dried in a vacuum oven at 80 °C for 8 h to remove the residual water. Acetyl tributyl citrate (ATBC-food grade) was supplied by Chou Feng Enterprise Co., Ltd. (Shulin, Taiwan). Dried components of PLA/starch at varying weight ratios were melt-blended using a Brabender. Three compounds were evaluated: PLA*_x_*Tapioca*_y_*, PLA*_x_*Tapioca*_y_*MDI and (PLA*_x_*Tapioca*_y_*MDI)*_a_*ATBC*_b_*. During each compounding process, the Brabender was operated at a temperature of 190 °C and a screw speed of 120 rpm for 3 min for all samples without ATBC and for an additional 2 min after adding ATBC. All prepared series were then hot-pressed at 190 °C and 10 MPa for 2 min and then cooled in air at about 25 °C. The compositions of all specimens are summarized in Table 1. Before hot-pressing, the specimens were dried in a vacuum oven at 80 °C for 12 h.

**Table 1:** Compositions of PLA, PLA*_x_*tapioca*_y_*, PLA*_x_*tapioca*_y_*MDI and (PLA*_x_*tapioca*_y_*MDI)*_a_*ATBC*_b_* specimens.

Sample	PLA (%)	Tapioca (%)	ATBC (%)	MDI (phr)
PLA	100	0	0	0
PLA_90_tapioca_10_	90	10	0	0
PLA_8__0_tapioca_2__0_	80	20	0	0
PLA_7__0_tapioca_3__0_	70	30	0	0
PLA_60_tapioca_40_	60	40	0	0
PLA_50_tapioca_5__0_	50	50	0	0
PLA_40_tapioca_6__0_	0	60	0	0
PLA_90_tapioca_10_MDI	90	10	0	0.5
PLA_8__0_tapioca_2__0_MDI	80	20	0	0.5
PLA_7__0_tapioca_3__0_MDI	70	30	0	0.5
PLA_60_tapioca_40_MDI	60	40	0	0.5
PLA_50_tapioca_5__0_MDI	50	50	0	0.5
PLA_60_tapioca_4__0_MDI	40	60	0	0.5
(PLA_90_tapioca_10_MDI)_95_ATBC_5_	85.5	9.5	5	0.5
(PLA_8__0_tapioca_2__0_MDI)_95_ATBC_5_	76	19	5	0.5
(PLA_7__0_tapioca_3__0_MDI)_95_ATBC_5_	66.5	28.5	5	0.5
(PLA_6__0_tapioca_4__0_MDI)_95_ATBC_5_	57	38	5	0.5
(PLA_5__0_tapioca_50_MDI)_95_ATBC_5_	47.5	47.5	5	0.5
(PLA_90_tapioca_10_MDI)_90_ATBC_10_	81	9	10	0.5
(PLA_8__0_tapioca_2__0_MDI)_90_ATBC_10_	72	18	10	0.5
(PLA_7__0_tapioca_3__0_MDI)_90_ATBC_10_	63	27	10	0.5
(PLA_6__0_tapioca_4__0_MDI)_90_ATBC_10_	54	36	10	0.5
(PLA_5__0_tapioca_50_MDI)_90_ATBC_10_	45	45	10	0.5
(PLA_90_tapioca_10_MDI)_85_ATBC_15_	76.5	8.5	15	0.5
(PLA_8__0_tapioca_2__0_MDI)_85_ATBC_15_	68	17	15	0.5
(PLA_7__0_tapioca_3__0_MDI)_85_ATBC_15_	59.5	25.5	15	0.5
(PLA_6__0_tapioca_4__0_MDI)_85_ATBC_15_	51	34	15	0.5
(PLA_5__0_tapioca_50_MDI)_85_ATBC_15_	42.5	42.5	15	0.5
(PLA_90_tapioca_10_MDI)_80_ATBC_20_	72	8	20	0.5
(PLA_8__0_tapioca_2__0_MDI)_80_ATBC_20_	64	16	20	0.5
(PLA_7__0_tapioca_3__0_MDI)_80_ATBC_20_	56	24	20	0.5
(PLA_6__0_tapioca_4__0_MDI)_80_ATBC_20_	48	32	20	0.5
(PLA_5__0_tapioca_50_MDI)_80_ATBC_20_	40	40	20	0.5
(PLA_90_tapioca_10_MDI)_75_ATBC_25_	67.5	7.5	25	0.5
(PLA_8__0_tapioca_2__0_MDI)_75_ATBC_25_	60	15	25	0.5
(PLA_7__0_tapioca_3__0_MDI)_75_ATBC_25_	52.2	22.5	25	0.5
(PLA_6__0_tapioca_4__0_MDI)_75_ATBC_25_	45	30	25	0.5
(PLA_5__0_tapioca_50_MDI)_75_ATBC_25_	37.5	37.5	25	0.5

### 3.2. Tensile Property

The tensile properties of the hot-pressed PLA, PLA*_x_*Tapioca*_y_* and (PLA*_x_*Tapioca*_y_*)/ATBC specimens at 25 °C were determined using a tensile testing machine (model AG-10KNA, Shimadzu Corporation, Kyoto, Japan) with a crosshead speed of 50 mm/min. A 35-mm gauge length was used for each tensile experiment. Dog-bone-shaped specimens were prepared according to the ASTM D638 Type IV standard [52]. On the basis of the average tensile results of at least five tensile specimens, the values of tensile strength and elongation at break were obtained.

### 3.3. Fourier Transform Infra-Red Spectroscopy

FTIR measurements were performed on a PerkinElmer spectrometer (model Spectrum One, PerkinElmer Inc., Waltham, MA, USA). The spectra of the samples were obtained by averaging 15 scans, with a wavenumber range of 4000 to 650 cm^−1^ and a resolution of 2 cm^−1^.

### 3.4. Morphology Analysis

The morphology of specimens, before and after the hydrolytic degradation, was observed by using a scanning electron microscope (model SU1510, Hitachi High-Technologies Corporation, Tokyo, Japan). Specimens of a 2 × 2 cm^2^ area were fixed on a sample holder using a conductive adhesive tape. They were coated with a thin layer of gold at 15 keV for 15 s to improve the image resolution and were then photographed at 3.00 K magnification and a low voltage of 2.1 kV.

### 3.5. Differential Scanning Calorimetry

The thermal properties of PLA composite resins were determined using a TA Q100 differential scanning calorimetry (DSC). All DSC scans were performed at a heating rate of 10 °C/min and under flowing nitrogen with a flow rate of 50 mL/min. The DSC was calibrated using pure indium. For *T*_g_ and *T*_m_ determination, samples weighing approximately 0.5 mg were placed in standard aluminum-sample pans.

### 3.6. Water Absorption

Five specimens (10 mm × 10 mm × 0.5 mm) were used for the water absorption test. After conditioning in desiccators for three weeks, specimens were weighed. They were immersed in distilled water at room temperature for 24 h. Then, they were dabbed with tissue paper to remove the water from the surface. Water absorption was calculated using the following Equation (1):

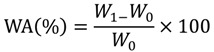
(1)
where *W*_0_ is the weight of the dry sample and *W*_1_ is the weight of the sample immersed in distilled water for 24 h.

### 3.7. Enzymatic Hydrolysis

The degradation of the enzymatic hydrolysis of specimens was evaluated at 27 °C using 50 mg starch enzyme in (0.025 mol Na_2_HPO_4_ + 0.025 mol KH_2_PO_4_) aqueous solution. Specimens with a dimension of 5 × 5 cm^2^ were tested for various days, washed with distilled water and dried completely in a vacuum oven at 70 °C for 8 h. On the basis of weight loss, the degree of degradation was determined using Equation (2):

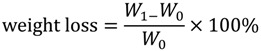
(2)
where *W*_0_ is the dry weight before degradation and *W*_t_ is the dry weight at time *t*.

## 4. Conclusions

The σ_f_ of PLA*_x_*tapioca*_y_*MDI specimens was significantly higher than that of PLA*_x_*tapioca*_y_* specimens. The ε_f_ of PLA and PLA*_x_*tapioca*_y_*MDI specimens approached the maximum value, as the ATBC content reached an optimum value of 10 and 15 wt%, respectively. The threshold limits of the ε_f_ were high when the ATBC content was 10 wt%. FTIR demonstrated the disappearance of the 3000 to 3670 cm^−1^ bending absorption band and the appearance of the 3315 and 1550 cm^−1^ NH stretching absorption band, which were attributed to the reaction of the OH groups of tapioca molecules with the N=C=O groups of MDI and/or to the reaction of the C-O-O-H groups of PLA molecules with the urethane groups of MDI during the melt-blending of PLA*_x_*tapioca*_y_* specimens. SEM micrographs revealed the intervals between PLA and tapioca. Voids from the matrix of PLA*_x_*tapioca*_y_* were significantly improved after MDI was added. Furthermore, two phases can be seen after the ATBC content reached 10 wt%. This is due to the exudation of ATBC; with increasing ATBC content, more demarcated plastic deformation was found on the surface of PLA_70_Tapioca_30_MDI. DSC curves of the PLA_70_tapioca_30_MDI specimen showed a single glass transition and cold crystallization that decreased as the ATBC content increased from 0 to 25 wt%. The increasing trend of water absorption with increasing ATBC content was attributed to the increasing free volume in PLA, causing the water molecules to be easily absorbed in the PLA*_x_*tapioca*_y_* specimens. Enzymatic hydrolysis tests indicated that the weight loss of PLA_70_tapioca_30_MDI increased significantly as the ATBC content increased.
